# Editorial: One- and Two-Dimensional Nanostructures for Drug Delivery Applications

**DOI:** 10.3389/fbioe.2021.782615

**Published:** 2021-10-13

**Authors:** Gozde Ozaydin Ince, Anna Maria Coclite, Barbara Stella

**Affiliations:** ^1^ Faculty of Engineering and Natural Sciences, Sabanci University, Istanbul, Turkey; ^2^ Institute of Solid State Physics, Graz University of Technology, Graz, Austria; ^3^ Dipartimento di Scienza e Tecnologia Del Farmaco, Università Degli Studi di Torino, Turin, Italy

**Keywords:** drug delivery systems, nanotubes, nanorods, nanofibers, thin films

**Editorial on the Research Topic**

**One- and Two-Dimensional Nanostructures for Drug Delivery Applications** Site specific and targeted delivery of therapeutic agents to specific body parts for the treatment of diseases is essential to increase drug efficacy and reduce toxic side effects. Employing specially designed nanoparticles as carriers of these therapeutic agents has proven effective for transporting drugs to the target and releasing when needed. Targeting is achieved by binding ligands to the nanoparticle surface to increase their uptake by the cells, enabling the targeting of different cells by changing the ligands used. Triggered release, which allows for drug release only at the target site, is achieved by using functional materials as the carrier particles that respond to changes in the target environment. Spherical nanoparticles are the most widely used nanostructures due to their relatively simple synthesis. However, spherical nanoparticles suffer from low drug loading capacities. Compared to zero dimensional nanoparticles (i.e., spherical nanoparticles), one-dimensional nanostructures (i.e., nanorods, nanofibers or nanotubes) have the advantages of high loading capacities and enhanced targeting due to their high surface to volume ratios. However, compared to spherical nanoparticles, one-dimensional nanostructures have been explored less as drug carriers, which can be attributed to their relatively challenging fabrication methods.

Longer circulation times, in addition to the alignment or tumbling of one-dimensional nanostructures during transport in the body, strongly impact the performance of these nanostructures as drug carriers. Although there are studies modeling the diffusion of these nanostructures in blood vessels, papers addressing the effect of their shape on the migration and transport mechanisms are limited. Furthermore, the effect of their shape on the uptake efficiency by the cells needs to be further explored. Similarly, two dimensional nanostructures have gained less attention compared to zero dimensional nanoparticles due to challenges in fabrication and severe immune response. However, recent studies have shown their significant benefits for particular treatments, such as multi-responsive control mechanisms and higher drug loading capacities. Therefore, this Research Topic invited researchers to contribute studies on recent advances in one- and two-dimensional nanostructures for drug delivery. We have collected three original research articles and one review, as reported below.

Yeniyurt et al. in their publication present the functionalization of single-wall carbon nanotubes (SWNTs) with various Fmoc-amino acid-bearing polyethylene glycol (PEG) chains of different length by a non-covalent method. First, molecular dynamics studies allowed suitable Fmoc-amino acids to be selected for an effective surface coating of SWNTs; afterwards, in experimental studies the Fmoc-amino acids were associated to SWNTs and these carriers were then characterized. The effect of modified SWNTs on the viability of human dermal fibroblast cells was also evaluated.

In another study, Balmori et al. address the important issue of protein corona formation on the surface of the nanoparticles used for cell-targeted delivery and investigated the effects of nanoparticle properties on the protein corona. The proteins adsorbing on the nanoparticle surfaces in the blood stream form a protein coating on the surface affecting the overall efficacy of the nanoparticles as cell targeting delivery devices. For this reason, a clear understanding of the interaction between the proteins and the nanoparticle surface is critical. Balmori et al. used Isothermal Titration Calorimetry (ITC) in combination with Nano Differential Scanning Calorimetry to study the effects of nanoparticle roughness and porosity on the adsorption of proteins to the particle surface. With the help of these two techniques, they could provide further explanation to the formation of hard protein corona formation inside the pores of the nanoparticles.

Khlyustova and Yang in their paper describe the kinetics of deposition of poly (4-aminostyrene) (PAS) thin films by initiated chemical vapor deposition (iCVD). PAS thin films can be used as 2D-nanomaterials for drug delivery, since the primary amine functional groups can react with biomolecules that enable targeted delivery or biocompatibility. In addition, the iCVD technique allows to deposite PAS thin films uniformly over drugs enabling their effective encapsulation. Understanding kinetics of PAS polymerization in iCVD is crucial for such deployments, because drug release kinetics in thin-film encapsulation have been shown to be determined by the film thickness. The authors apply a dual-regime polymerization model to explain the growth rate as a function of fractional saturation pressure of the monomer: they demonstrate that at the critical fractional saturation pressure value of 0.2, a transition from quadratic dependence to linear dependence takes place.

Gleason reviews the applications of polymeric nanolayers grown by iCVD for the controlled release of pharmaceuticals and other molecules. Starting from the description of the composition and the design of different iCVD polymers, the author describes the physico-chemical properties that they should possess for controlled release applications. The iCVD polymerization process is described in details and the characterization techniques are also reported. Finally, the paper reviews the wide range of structures that have been encapsulated by iCVD for controlled release of drugs, such as membranes, micro- and nanoparticles, fibers, textiles and porous media.

In summary, this Research Topic covers recent advances in the field of one- and two-dimensional nanostructures for drug delivery, providing new insights into effects of nanostructures on release kinetics and immune response to these nanostructures. The editors hope that the Research Topic “*One- and Two-Dimensional Nanostructures for Drug Delivery Applications*” will contribute to the research field of drug delivery and increase the interest on one- and two-dimensional nanostructures as drug carrier systems.

